# Genomic and oncoproteomic advances in detection and treatment of colorectal cancer

**DOI:** 10.1186/1477-7819-7-36

**Published:** 2009-04-01

**Authors:** Seamus M McHugh, Jill O'Donnell, Peter Gillen

**Affiliations:** 1 Dept. of Surgery, Our Lady of Lourdes Hospital, Drogheda, County Louth, Ireland

## Abstract

**Aims:**

We will examine the latest advances in genomic and proteomic laboratory technology. Through an extensive literature review we aim to critically appraise those studies which have utilized these latest technologies and ascertain their potential to identify clinically useful biomarkers.

**Methods:**

An extensive review of the literature was carried out in both online medical journals and through the Royal College of Surgeons in Ireland library.

**Results:**

Laboratory technology has advanced in the fields of genomics and oncoproteomics. Gene expression profiling with DNA microarray technology has allowed us to begin genetic profiling of colorectal cancer tissue. The response to chemotherapy can differ amongst individual tumors. For the first time researchers have begun to isolate and identify the genes responsible. New laboratory techniques allow us to isolate proteins preferentially expressed in colorectal cancer tissue. This could potentially lead to identification of a clinically useful protein biomarker in colorectal cancer screening and treatment.

**Conclusion:**

If a set of discriminating genes could be used for characterization and prediction of chemotherapeutic response, an individualized tailored therapeutic regime could become the standard of care for those undergoing systemic treatment for colorectal cancer. New laboratory techniques of protein identification may eventually allow identification of a clinically useful biomarker that could be used for screening and treatment. At present however, both expression of different gene signatures and isolation of various protein peaks has been limited by study size. Independent multi-centre correlation of results with larger sample sizes is needed to allow translation into clinical practice.

## Background

Colorectal cancer (CRC) is the most abundant type of neoplasia in developed countries, and the second cause of death among cancers [1]. Understanding the molecular basis of the biochemical pathways involved in carcinogenesis can facilitate diagnosis and treatment of cancer. Current knowledge of cellular regulation indicates that many networks operate at the epigenetic, transcriptional and translational levels. Genomic and proteomic technologies will help further understand the intracellular signaling and gene transcription systems as well as the protein pathways that connect extracellular microenvironment to the serum or plasma macroenvironment [2].

Initial genomic studies focused on changes in global expression levels, using microarray or serial analysis of gene expression analysis [3]. Gene expression is the process in which the inheritable information in a gene, such as the DNA sequence, is made into a functional gene product, such as protein or RNA. DNA microarrays allow us to visualize the expression of potentially all genes within a cell population or tissue sample. The analysis of this type of data is commonly called gene expression profiling. New advances in genomic techniques such as DNA microarray analysis may make possible the identification of patients who will respond to adjuvant therapy. This could individualise treatment regimes and avoid unnecessary treatments in those deemed non-responders.

Genomics is also being used to search for a novel CRC biomarker. Because CRC develops slowly via a progressive accumulation of genetic mutations, recurrence rates and overall mortality due to CRC is closely related to the stage of disease at time of diagnosis [4]. Evidence exists to suggest endoscopic screening by sigmoidoscopy reduces incidence of distal CRC [5] and subsequent death. However, despite the different available screening methods and their proven benefits, morbidity and mortality associated with CRC remains high, partly due to a low compliance with screening [6]. If a novel biomarker that could be used to detect CRC early were to be developed, this would have far reaching benefits both for the individual and for health services as a whole. Microarray analysis of colonocytes, which are shed into the faecal stream, can be used to detect genetic markers for CRC in faeces. Genomic examination of DNA methylation has also highlighted genes that could potentially serve as molecular biomarkers.

Genomic advances aside, recent literature published in the field of oncoproteomics also highlights potential novel biomarkers to aid in the early detection of colorectal cancer. Alterations in protein abundance, structure or function can act as indicators of carcinogenesis prior to development of clinical symptoms [7]. Currently carcinoembryonic antigen (CEA) is the best characterised serological marker for CRC. However European guidelines limit its use to the detection of recurrence for patients with stage II or III who may be candidates for either liver resection or systemic therapy should recurrence develop [8]. Advances in protemic techniques and analytical techniques in mass spectrometry provide greater opportunity to isolate individual peptides that could be used to detect CRC at an early stage.

### Genomics

#### Genomics and response to chemotherapy

Radical resection is the main treatment for adenocarcinoma of the colon. However, 50% of patients with diagnosed colorectal carcinoma develop liver metastasis at some point during their lifetime [9]. The response to chemotherapy differs amongst individual tumours [10,11]. If a set of discriminating genes could be used for characterisation and prediction of response, an individualised tailored therapeutic regime could become the standard of care for those undergoing systemic treatment for CRC. Numerous molecular markers have been studied in those undergoing adjuvant therapies. Epidermal growth factor receptor (EGRF) expression after chemotherapy has been associated with disease free survival, and expression of p21 along with MIB-1 after neoadjuvant chemoradiotherapy predicts a worse outcome [12].

Advances in gene expression are producing studies which claim increased efficiency in predicting response to 5-Fluourouracil(5-FU)-induced apoptosis [13]. In 1991 preferential use of the orotate phosphoribosyl transferase (OPRT) metabolic pathway in the metabolism of 5-FU was shown to correlate with higher chemosensitivity in CRC tissue [14]. Gene expression profiling had again highlighted it's potential as a predictor of response to 5-FU [15]. A recent study investigated the prognostic value of the expression of the 5-FU metabolic enzyme genes, including OPRT in 103 CRC patients (Duke's stage B and C) treated with oral 5-FU-based adjuvant chemotherapy [16]. It found that the disease-free and overall survival of the OPRT mRNA high-expression group were significantly longer than that of the OPRT mRNA low-expression group.

For CRC patients being treated with leucovorin, fluorouracil and irinotecan (FOLFIRI), small in vivo studies have isolated a set of 14 predictor genes of response with 100% specificity and 92% sensitivity [17]. Here 40 patients with synchronous and unresectable liver metastases underwent primary tumour resection and adjuvant chemotherapy. The 14 genes over expressed in responder tumours were functionally classed as RNA splicing genes, regulation of transcription, cell adhesion, cell differentiation, ion transport, signal transduction, development, visual perception, and a Golgi membrane protein gene. These genes were all over expressed in the responder group to FOLFIRI. However these results are based on a small sample size. Further studies of these 14 genes are necessary in a larger independent cohort of patients. This study was the first predictor classifier based on microarray gene expression in CRC. Since it's publication there have been several further studies [18-20], but many of these have yet to show consistency with regard to the gene signature being studied.

UDP glucoronosyltransferase 1 (UGT1A1) is a gene which encodes an enzyme of the glucuronidation pathway. It's variations have been examined in metastatic CRC patients treated with irinotecan. A recent evidence-based review described the proposed clinical utility of UGT1A1 genotyping, as three recent studies they reviewed found statistically significant higher tumour response rates among individuals homozygous for a particular allele [21]. They concluded that a prospective RCT was necessary to examine the effects of irinotecan dose modification in CRC patients based on their UGT1A1 genotype. This could improve tumour response in CRC cancer patients with various UGT1A1 genotypes, as well as minimising unnecessary adverse reactions such as severe neutropaenia.

The first report using DNA microarray for predicting response to radiotherapy was published in 2006 [22]. Japanese researchers identified a novel set of 33 discriminating genes that could predict responders and non-responders to preoperative radiotherapy in rectal cancer. Because the number of patients – especially responders – was limited, larger prospective trials will be needed to confirm results.

The role of a PTMA (prothymosin alpha), a gene considered to have a nuclear function related to cell proliferation was investigated recently [23]. In this study, PTMA was found to be upregulated in radiotherapy resistant CRC. The researchers analysed clinical samples from 30 irradiated rectal cancer patients. The expression of PTMA was found to be statistically significantly higher in radioresistant patients. PTMA expression was only significantly upregulated in irradiated tissue. Further studies investigating it's expression in CRC tissue prior to radiotherapy are needed in order to ascertain it's effectiveness as a predictor of response. Only prediction of non-responders without their having to undergo any unnecessary radiation would be clinically useful.

A team of Korean researchers investigated whether microarray gene expression analysis could predict complete response to preoperative chemoradiotherapy in rectal cancer [24]. In their study, 46 patients (31 for training and 15 for validation testing) with rectal carcinoma underwent preoperative RCT and surgical excision 6 weeks later. Baseline tissue samples were collected prior to treatment. After excision, the tumour samples were classified as complete or partial responders to RCT using Dworak's tumour regression grade system [25]. Using microarray analysis 261 genes were identified as differing between the two groups, with the 95 top ranked of these predictor genes being able to distinguish between partial and complete responders in 84% of 31 training samples and 87% of the validation samples.

Another study looking to use genomics to predict response of CRC patients to chemoradiotherapy was published recently [26]. The study constructed gene expression profiles of 43 biopsy specimens of locally advanced rectal carcinomas to identify 42 genes that could differentiate responders from non-responders. These genes were mostly encoding proteins that played a role in the nucleus, such as the transcription factor ETS2, or were associated with transport function, such as the solute carrier SLC35E1 or the regulation of apoptosis, such as caspase-1.

Establishing validated molecular analysis and subsequent tumour gene-signature identification allows patients with early stage cancer with low recurrence risk to be spared the toxicity of systemic chemotherapy and/or radiotherapy. In addition patients identified as non-responders would be spared unnecessary side effects. From an economic perspective this would have huge benefits. However the transfer from laboratory to bedside is proving more laborious than expected. More independent laboratories need to examine the same gene signatures. At present there is a lack of consistency with different studies all producing results but using different sets of genes, and often with small numbers.

#### Potential genetic biomarkers

Identification of genes characteristic CRC development could uncover biomarkers which would aid in CRC diagnosis and screening. Faecal-occult-blood testing (FOBT) is currently the most widely used screening modality for CRC. However it has poor sensitivity for detection of CRC, with large randomised clinical trials showing only a 30% reduction in mortality [27]. Colonocytes, which are shed continuously and with greater frequency from CRC tissue than normal colonic mucosa have been analysed for genetic mutations. Several genes have thus been isolated as potential markers for CRC. p53 and adenomatous polyposis coli (APC) both genetically encode tumour suppressor proteins which regulate apoptosis and angiogenesis. Although up to 60% of CRCs demonstrate p53 mutations [28], these appear late in the genesis of CRC and so have limited use in it's early detection. In contrast, APC appears to be an early genetic event in the development of CRC. However the mutations are distributed throughout the coding region of DNA making it difficult to detect all mutations in screening for CRC [4].

Cancer specific or "type C" DNA methylation has been shown to lead to transcriptional silencing of various genes such as tumour suppressor genes and genes involved in DNA repair and apoptosis. When a large number of CRCs were examined, some were found to accumulate high frequencies of type C methylation of multiple genes. This subset of CRC tumours is classified as having CpG island methylator phenotype. Using DNA microanalysis identification of a number of genes that are epigenetically silenced in colorectal cancer has been made possible.

One in-vitro using tissue from 124 tumours highlighted the SFRP gene [29]. It reported that hypermethylation of the four genes in this family occurs with high frequency in CRC, potentially providing for construction of molecular marker panel for CRC detection. A further in-vitro study described high incidence of BRAF mutations and micro-satellite instability (MSI) in a group of tumours with high methylation frequency [30].

Most recently, oncostatin M (OSM), a member of the interleukin-6 cytokine family has been examined. This gene family inhibits cell proliferation and induces apoptosis in cancers. The OSM-receptor in CRC was studied in a recent publication [31]. In this study of 98 CRCs, silencing of the OSM-receptor by methylation was observed in 90% of cases.

Studies involving DNA methylation have highlighted several genes which play an important part in CRC carcinogenesis. Their potential for development into molecular markers for early CRC diagnosis is evident. However despite these findings, conflicting reports exist associating type C methylation with normal aging, or with microsatellite instability rather than carcinomatous change [32].

Recent advances in genomic technology such as ultra-high-throughput microarray analysis allow us describe previously inaccessible components of the genome. Although it has been used to identify tumour suppressor genes in patients with multiple myeloma, it has yet to be applied to colorectal cancer [33].

The pathway to develop a clinically useful biomarker from a potential gene identified is a long one, and further correlative studies are needed to cement and develop the genetic associations highlighted in these recent publications.

Other potential biomarkers can be may be isolated through advances in proteomics. Protein biomarkers are based on aberrant protein signalling circuits represented by post-translational modifications. As such proteomics could be expected to render better insight than genomics with regard to developing a biomarker for screening for disease screening, progression and treatment response [34].

### Oncoproteomics

#### 2-DE

To date the primary technique for proteomic biomarker discovery has been Two Dimensional Electrophoresis (2-DE). Using this method subcellular fractions are separated by charge and then by molecular weight. These proteins are mixed on a gel then scanned to generate a map for each labelled protein. Maps from different patient samples can then be compared to ascertain which proteins are expressed in one sample and not another [35]. However, the comparison between two different gel samples remains difficult. Each gel runs slightly differently, which makes gel-to-gel comparison laborious. Recently, 2D difference-in-gel electrophoresis (DIGE) has been introduced. This technique minimises gel-to-gel variations [36]. However the exchange of 2-DIGE data between laboratories has been a problem due to spatial irreproducibility between 2D gels generated [37].

Standing alone 2-DE is purely a descriptive technique and as such must be coupled with analytical methods such as Mass Spectrometry (MS). Proteins are extracted from the 2-DE gel and characterised for protein identification using structural information such as peptide mass or amino acid sequence. These values are checked against a known database and the proteins thus identified.

2-DE was first used to study protein profiles in carcinoma cells by the Gottesman's group as early as 1986 [38]. Several publications have investigated the utility of 2-DE in CRC, with the idea of identifying a clinical biomarker using proteomics in it's infancy [39-43], and recent studies have identified potential biomarkers which might be used to screen for CRC.

One such study demonstrated the down-regulation of secretagogin, a protein expressed in neuroendocrine cells of the colonic crypts in carcinomatous mucosal cells involved in calcium-binding. The study concluded that expression of secretagogin in non-neuronal and non-neuroendocrine cells may represent aberrant expression of the protein and may be related to de- or trans-differentiation phenomena. This was an invitro study using immunohistochemistry, and so further in vivo studies are needed for it to progress to a clinical setting [44]. However, it has certainly been highlighted as a potential for the future. Not alone implicated in CRC, secretagogin expression is currently under scrutiny in several tumour types, with recent studies examining it's role in prostatic adenocarcinoma, pituitary adenomas, carcinoid tumours and their metastases as well as neuroendocrine tumours from the lung, pancreas and adrenal gland [45-47]. In a study published by the department of Neurosurgery in Vienna, expression of secretagogin in endothelial cells of blood vessels in some meningiomas, haemangiopericytomas and haemangioblastomas led to the theory that it is implicated in angiogenic activity in human cancer [48].

Further 2-DE studies include a piece from Singapore in 2006 which examined 7 pairs of samples (one of the pair from CRC tissue, the other from adjacent normal tissue) from 7 patients with diagnosed stage 3 CRC [49]. In this study, DIGE was used to compare between gel samples. Here, glycolytic enzyme proteins were demonstrated to be up regulated in the tumour samples. Mirroring this, phosphoenolpyruvate carboxylase, a key regulatory enzyme in gluoneogenesis was found to be down regulated. Also down regulated were enzymes at the early entrance of the tricarboxylic acid cycle, suggesting it's impairment in tumour cells. These extensive alterations in metabolic pathways have potential for design of novel biomarkers. Unwin et al [50] were the first to demonstrate by proteomics that the glycolytic pathway was elevated in renal cancer tissue (named "The Warburg effect"). A subsequent study supported this observation in 24 classes of cancer tissue [51]. But it remains controversial whether the increase of glycolytic activity is due to inherent metabolic alterations at all. It may simply be secondary to the anaerobic environment of tumour tissue [52].

#### MALDI-TOF

In addition to 2-DE, promising new methods are now being used in the search for a new biomarker. Using Matrix-Assisted Laser Desorption/Ionization – Time of Flight technique (MALDI-TOF) the sample to be analysed is mixed with an energy absorbing matrix molecule which absorbs light at a predetermined wavelength. The sample is irradiated with a laser to convert the crystalline matrix to a gas, and peptide ions are ejected from the target surface. They can then be directed down a vacuum chamber and separated based on their time of flight. These different times of flights for different proteins are then used to generate a 3 dimensional algorhythm, which can have several thousand data points, with particular protein ion clusters being evident as graph peaks.

As well as a small recent in-vivo study identifying proteins overly expressed in CRC cells as compared to normal colonic mucosa [53], MALDI-TOF has now been used to differentiate CRC patients from healthy controls [54]. In a randomised block design, pre-operative serum samples obtained from 66 colorectal cancer patients and 50 controls were used to generate high-resolution MALDI-TOF protein profiles. Thirty-four patients out of thirty-seven with early stage disease (stage 1 and 2) and all patients with stage 3 or 4 disease were correctly classified as having cancer. As a confounder however, there was significant difference in age between groups, with the control group being younger than the CRC patients. Also, because of small sample size, a further independent validation study would be necessary to add weight to these findings.

MALDI-TOF technology is also being applied in the search to predict metastasis in known cases of CRC. Two CRC cell lines with different metastatic potentials, SW480 and SW620, were recently investigated using MALDI-TOF to search for potential markers for predicting CRC metastasis. Heat Shock Protein (Hsp) 27 overexpression was found to relate to metastatic behaviour in a CRC cell [55]. Hsp27 is a cytoprotective chaperone that is phosphoactivated during cell stress that prevents aggregation and/or regulates activity and degradation of certain client proteins. For more than 10 years, HSP 27 has been under the spotlight for it's role in carcinogenesis [56-59] and it has also recently been implicated in irinotecan resistance in CRC [60]. However in these cell lines such results are only stepping stones in the formation of larger in-vitro studies necessary.

A further MALDI-TOF study targeted T Lymphoma invasion and metastasis 1 (Tiam 1), a guanine nucleotide exchange factor that activates Rac (a GTPase responsible for stimulating cell spreading and migration). Having found that Tiam1 was highly related to the metastatic potential of CRC [61], the team then used the MALDI-TOF technology to identify 11 differentially expressed proteins were identified in the CRC HT29 cell line transfected with Tiam [62]. The identification of these down-stream targets of Tiam1 (one of which included Hsp 27) may eventually allow clinicians to identify CRC patients at high risk of metastasis.

#### SELDI-TOF

Surface enhanced laser desorption ionization/time of flight (SELDI-TOF) is a new method of complex protein lysis based on MALDI technology. Using SELDI, the proteins from a given sample are selectively retained on a platform using chemical or biological agent. This selective retention based upon intrinsic peptide properties allows for the isolation and subsequent analysis of less abundant proteins. As in MALDI-TOF, ionisation again occurs using laser emission, and the peptide ions thus formed then guided into the MS analyser.

The first SELDI-TOF study attempted to differentiate CRC patients from those with colorectal adenoma. Seven protein peaks were isolated as potential biomarkers, but unfortunately these were not specific to CRC [63].

Another study comprised of two sets of samples [64]. The first samples were from 40 CRC patients (all Dukes' D) and 49 controls. The second set consisted of samples from 37 CRC patients and 31 healthy controls. They reported three potential biomarkers with a sensitivity and specificity between 65% and 90%. A further study lent weight to the theory that SELDI-TOF could be used to distinguish CRC patients from healthy controls [65]. The major failing in these studies is their investigation of unrelated proteins. Multiple studies of the same protein peaks producing similar results are needed before transition to clinical practice can occur.

SELDI-TOF is not without it's limitations. As with any other analytical technique, not all proteins can be visualised well. Sensitivity for higher molecular weight proteins is lower than for those in the less than 20 kDa range. Also creating a reliable protein profile from biological samples remains a problem, as in many cases mass resolution is found to be too low. This makes data comparison and verification between laboratories difficult [66].

SELDI-TOF technology is has recently been applied to the identification of responders and non-responders to neo adjuvant chemoradiotherapy (RCT). A study by Smith F.M. et al [67] used SELDI-TOF MS to identify 14 protein peaks from serum samples taken 24–48 hours post commencement of RCT in 20 patients with rectal cancer. While there was no significant difference in baseline protein peaks, protein peaks at 24 hours post beginning RCT were significant. As such this study claims that these isolated protein peaks may potentially be used to determine responders from non-responders to RCT, but not without their undergoing RCT initially.

Granted avoiding unnecessary RCT complications in non-responders would be a noteworthy achievement, but even more noteworthy would be the sensitisation of these drug resistant patients to their chemo-therapeutic agent. In a recent review Zhang J-T et al mention several mechanisms of resistance only recently discovered using proteomic technology [68]. Notable different proteins associated with chemotherapy drug resistance in CRC highlighted in this review included Hsp27 (described previously), Anexin IV (like secretagogin, another calcium-binding protein) as well as 14-3-3sigma (a protein involved in regulation of the cell cycle). The Anexin family of proteins have been investigated before for their role in carcinogenesis. There have already been studies of their expression in renal clear cell [69] and prostate carcinoma [70]. 14-3-3sigma has previously been investigated for it's role not only in CRC [71] but also in breast cancer and pancreatic adenocarcinoma [72,73].

Unfortunately it would be overly optimistic to hope that targeting these isolated proteins would increase patient sensitivity in non-responders since resistance of a given tumour to chemotherapeutic agents likely has multiple mechanisms of resistance [68]. Combination therapies targeting multiple proteins to sensitise the drug resistant patient is a goal to strive for in the future of cancer treatment, but technology has not advanced sufficiently to allow that yet.

#### Advances in mass spectrometry

Mass spectrometry (MS) has become the analytical tool of choice in proteomic study owing to it's quantitative capability and facility to interface with the different chromatographic separation methods.

The conventional pipeline for biomarker development involves a discovery phase, through advances in proteomic technology described above combined with MS followed by validation and clinical application, usually on an alternative platform, such as immunoassay. Though the most sensitive, the development of an immunoassay is time consuming when antibodies are not available and need to be conceived. Mass spectrometry analysis driven in quantitative multiple reaction monitoring (MRM) mode is now appearing as a promising alternative to quantify proteins in biological fluids. This mode conducts both biomarker discovery and validation on the same platform, thus obviating the need for parallel assay development [74]. This is both time saving and cost effective. In MRM, MS analysis time is focused only on analytes of specific masses, while all others are excluded. Fragmenting the analyte and monitoring both parent and one or more product ions simultaneously can also attain further specificity. The application of MRM to proteomic analysis has only recently been adopted because of advances in MS instrumentation. To date there are very few publications describing the use of MRM for detection of plasma biomarkers. These studies highlighted fibulin-2 as a breast cancer marker in mice [75], and CEA as a lung cancer marker [76]. It's application to CRC has yet to produce a definite potential biomarker.

Fourier transform ion cyclotron resonance (FT-ICR) instruments are currently used in proteome analysis to analyse proteins and peptides with high resolution and mass accuracy. In FT-ICR, ions from multiple laser shots are accumulated in a hexapole and then guided with a quadrupole ion field into the ICR cell where the ions cyclotron in a magnetic field. Ion frequencies are then measured, and these frequencies resolved into sinusoidal curves using fourier analysis. Unfortunately the high costs and complexity of these instruments limits their use [77]. A more compact less costly mass spectrometer has been developed in the Linear trap quadrupole (LTQ) Orbitrap. The LTQ Orbitrap consists of a spindle-like central electrode and a barrel-like outer electrode. When voltage is applied between the two, ions injected into the Orbitrap they experience a monotonic increase in electric field strength which contracts the radius of the ion cloud, thus decreasing the possibility of losing ions to collusions with the outer electrode [78]. This new analytical tool has high resolving power with good mass accuracy to reduce false positive peptide identifications. It has yet to be used to develop a CRC biomarker but such technological advances hold promise for protein identification with high specificity.

Absolute quantification (AQUA) is a method many laboratories use for MS-based biomarker validation [79]. In an AQUA study, a peptide containing a stable-isotope labeled amino acid is developed based on the sequence of a peptide that is being targeted for quantitation. This synthesized peptide is spiked into the complex proteome sample and used as an internal standard for quantitation purposes. Use of AQUA for validation of biomarkers tends to be less time consuming than MS-based quantitation of peptides. However each synthesized peptide needs to be manufactured individually, which makes concurrent quantitation of multiple peptides difficult.

Using the various technologies described here, MS-based discovery studies have identified a huge number of potential biomarkers for specific diseases. Presently, the focus is on developing MS-based MRM scanning methods to measure the absolute quantity of known proteins within complex clinical samples [78]. To further the discovery of a clinically effective biomarker there is a need for targeted quantitative methods of proteomic profiling and these new advances make this increasingly possible. However the cost of MS instruments combined with lack of highly specific antibodies for many proteins for MS-based biomarker validation methods still needs to be further addressed.

## Conclusion

These are all noteworthy discoveries, but are they viable for translation into everyday clinical practice? We must remember that the use of mass spectrometry to develop individual protein spectra is not in itself a realistically practical method of screening from a cost effective viewpoint. Rather it is a stepping-stone towards the development of a useful biomarker. Since screening for colorectal cancer is cost effective [79], if a simple blood biomarker for colorectal cancer could be developed it would have huge financial implications for health services worldwide.

There are many obstacles to overcome in the future application of proteomics and genomics in clinical practice. No solitary biomarker is considered adequately sensitive and specific for CRC screening. Rather it is expected that the results of multiple markers will need to be combined to yield accurate classification [80]. There remains a lack of clear guidelines for manufacturing and laboratory practice for all phases of biomarker development [81]. Quality control must be implemented to assure reproducibility and accuracy. To date there remains a lack of consistent investigation into specific gene signatures or protein peaks. Different studies of limited sizes have highlighted numerous potential biomarkers. There is not enough independent multi-centre correlation to confidently claim that identification of a biomarker is imminent. It is at least however possible. And with further advances in laboratory technology, and larger corroborative studies, it remains a goal for the future.

## Conflict of interests

The authors declare that they have no competing interests.

## Authors' contributions

SMM is the lead author and was involved in writing article review as well as revising manuscript in order to include any amendments suggested, and also undertook extensive literature review. JOD was involved in conception of article review subject matter and also undertook extensive literature review as well as editing early drafts of manuscript. PG was involved in conception of article review subject matter and also surpervised writing and editing of drafts of manuscript prior to submission for publication. All authors read and approved the final manuscript.
